# Laboratory Study of the Influence of Substrate Type and Temperature on the Exploratory Tunneling by Formosan Subterranean Termite

**DOI:** 10.3390/insects3030629

**Published:** 2012-06-27

**Authors:** Bal K. Gautam, Gregg Henderson

**Affiliations:** Department of Entomology, Louisiana State University AgCenter, Baton Rouge, LA 70802, USA; E-Mail: bgautam@agcenter.lsu.edu

**Keywords:** soil types, foraging, substrate preference, *Coptotermes formosanus*

## Abstract

Using two-dimensional foraging arenas, laboratory tests were conducted to investigate the effect of soil type, soil moisture level and ambient temperature on the exploratory tunneling by *Coptotermes formosanus* Shiraki. In choice arenas consisting of two substrate types having two moisture levels each, and conducted at a constant temperature of 22 °C, a significantly greater proportion of termites aggregated in sand than in sandy loam. Similarly, the length of excavated tunnels was also increased in sand. In a given substrate, termite aggregation or tunnel length did not differ between 5% and 15% moisture levels. In no-choice tests, where three different substrates (sand, sandy loam and silt loam) were tested at two temperatures (22 °C and 28 °C), excavations were significantly greater in sand than either sandy loam or silt loam at 22 °C. Fewer primary tunnels were constructed in sandy loam than in sand and fewer branched tunnels than either in sand or silt loam. No significant difference in either tunnel length or number of primary or branched tunnels was found between these two temperatures.

## 1. Introduction

Subterranean termites search for food by constructing a ramified tunnel and gallery system underneath and above the soil. Usually primary tunnels radiate from the nest and as they go farther away these main tunnels split into secondary and tertiary tunnels [1,2]. The efficiency of food finding by subterranean termites therefore depends on the organization of the tunnel systems [3]. Studies have shown that environmental factors such as soil texture, soil moisture, ambient temperature and the presence or absence of food sources influence the search tunnel formation by subterranean termites [4,5,6,7,8,9,10,11].

The presence and amount of food have been reported to influence the branching and total length of tunnel system constructed by subterranean termites such as *C. formosanus *[1] and *Reticulitermes santonensis* De Feytaud [12]. On the other hand, there are studies [13,14] which suggest that food search by *C. formosanus* is systematic and not affected by food in the foraging arena. Nevertheless, these studies agree that the main and branched tunnels are organized in a way that they avoid searching the area that was already explored. 

Although there is no debate on the requirement of substrate moisture in the foraging arena, there are still contradictory reports on the influence of a particular level of moisture in different substrates for search tunnel formation by subterranean termites. An early tunneling activity by *C. formosanus* was found to be greater in high moisture sand than in low moisture sand when provided with a moisture gradient [8]. Likewise, the tunneling rate by *C. frenchi* Hill was reported to be greater in wet sand than in dry sand [7] and *C. gestroi* (Wasmann) was found to construct more secondary tunnels at 15% moisture sand than in 5% moisture sand [2]. In contrast, Potter *et al.* demonstrated from their field study that there was no difference in damage ratings between Sentricon^®^ stations placed in wet soil or dry soil [15]. One confounding factor here is the use of ‘dry soil’ in the experiment. It is difficult to determine (the authors have not mentioned) whether the dry soil in these studies was completely dry or had low moisture levels, because the survival of subterranean termites is a major issue in a completely dry soil. When a wet food matrix is provided or when the dry soil is adjoining wet soil then termites can relocate water to make the dry soil moist [16,17]. Like moisture, ambient temperature also influences the tunneling behavior of subterranean termites. For example, *C. gestroi *and *Heterotermes tenuis* (Hagen) constructed more secondary tunnels at 20 °C and 25 °C than at 15 °C in sand [2]. 

Soil texture impacts subterranean termite foraging both by its particle size and its water holding properties. As the soil particles become coarser the water holding capacity of the soil decreases [18]; as a result, relatively more water is directly available to the termites. In sand, the moisture content as low as 4% by wt. provides sufficient moisture for *C. formosanus* to tunnel and aggregate normally [11]. A faster rate of tunneling by *C. formosanus *was reported in sand than in top soil and clay [10]. In moist conditions, however, *C. formosanus* preferred to aggregate in soil with finer particles than in soils with coarser particles and the opposite was found in dry condition [10]. *C. gestroi *excavated significantly more secondary tunnels in soil with uniform particle size of 2.0 mm than in soil with heterogeneous particle size (0.002–4.00 mm) while *H. tenuis *could not even penetrate the arenas filled with the latter soil type [2]. *C. formosanus* and *Reticulitermes hesperus* Banks constructed different tunneling patterns based on the soil particle size [5]. In this study, first we aimed to test the substrate preference between sand and sandy loam at two different moisture levels and second to compare the rate and pattern of tunneling among sand, sandy loam and silt loam at two ambient temperatures. 

## 2. Results and Discussion

**Figure 1:**
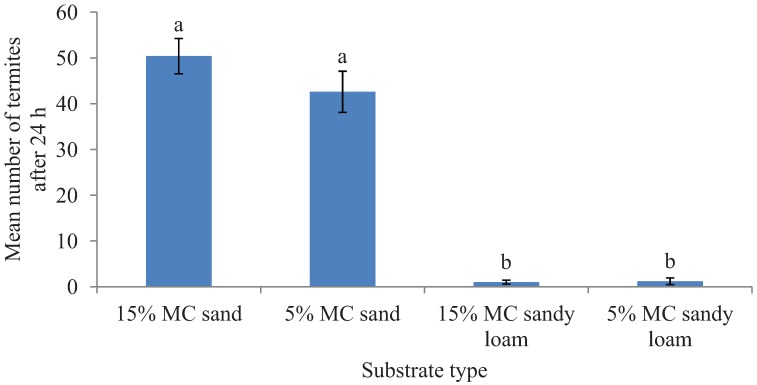
Mean number (±SEM) of termites recovered in two different substrates each with two moisture levels (MC) in the preference tests after 24 h period. Bars with different letters are significantly different (α < 0.05, Tukey’s test) from each other.

**Figure 2:**
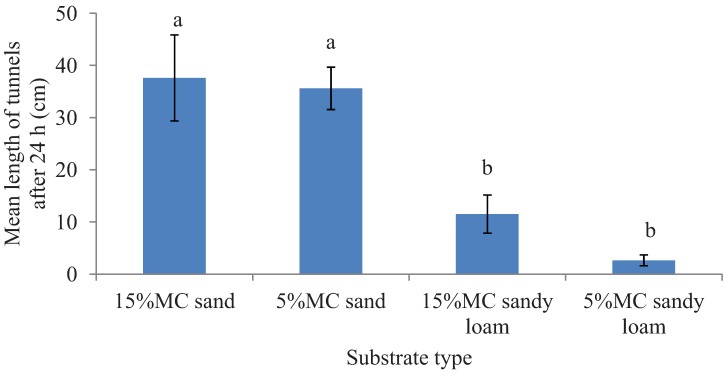
Mean length (±SEM) of tunnels excavated in two different substrates each with two moisture levels (MC) in the preference tests in 24 h period. Bars with different letters are significantly different (α < 0.05, Tukey’s test) from each other.

Termite mortality was insignificant in both the experiments as ≥96% termites were recovered active and live at the end of the bioassays (survival data for the preference test is given in Figure 1 but the survival data for tunneling test is not provided). In the preference tests, a significantly greater total length of tunnels was excavated in sand compared to sandy loam (*F* = 35.26; df = 1, 16; *p* < 0.0001) in the 24 h period. However, tunnel length was not significantly different between the two moisture levels (*F* = 1.19; df = 1, 16; *p* = 0.29) and there was no interaction effect of the substrates and the moisture levels (*F* = 0.48; df = 1, 16; *p* = 0.50) (Figure 2)*.* Likewise, after 24 h, a significantly greater proportion of released termites were recovered in sand than in the sandy loam (*F* = 325.37; df = 1, 16; *p* < 0.0001) but the tested moisture levels had no significant impact on the number of termites found on a given substrate type (*F* = 0.69; df = 1, 16; *p* = 0.43) and had no interaction effect of the substrate and moisture levels (*F* = 0.88; df = 1, 16; *p* = 0.36) (Figure 1).

In the tunneling tests, substrate type had a significant impact on the length of tunnels at both time periods (14 h post-release: *F* = 6.57; df = 2, 12; *p* = 0.0118 and 24 h post-release: *F* = 7.71; df = 2, 12; *p* = 0.0070). The length of tunnels was not significantly different between 22 °C and 28 °C (14 h post-release: *F* = 0.03; df = 1, 12; *p* = 0.8745 and 24 h post-release: *F* = 0.01; df = 1, 12; *p* = 0.9260), however, there was a substrate type and temperature interaction effect at 24 h post-release (14 h post-release: *F* = 3.77; df = 2, 12; *p* = 0.0538 and 24 h post-release: *F* = 5.28; df = 2, 12; *p* = 0.0227). Means comparison showed that there was significant difference in the lengths of tunnels among the substrates at 22 °C but not at 28 °C. At 14 h post-release, the length of tunnels was significantly greater in sand than in sandy loam and after 24 h post-release it was significantly greater in both the sand and the silt loam compared to sandy loam (Table 1). 

**Table 1:** Mean length (±SEM) of tunnels excavated in 14 h and 24 h periods in three different substrates at two temperature conditions.

Substrate	Mean length of tunnels in 14 h (cm)		Mean lengths of tunnels in 24 h (cm)	
	28 °C	22 °C	28 °C	22 °C
sand	80.00 ± 16.64 ^a^	99.36 ± 7.62 ^a^	104.83 ± 13.49 ^a^	123.16 ± 9.09 ^a^
sandy loam	66.83 ± 13.63 ^a^	28.53 ± 10.25 ^b^	86.73 ± 14.30 ^a^	39.90 ±13.73 ^b^
silt loam	58.00 ± 2.51 ^a^	72.33 ± 13.41 ^ab^	72.33 ± 2.42 ^a^	103.83 ± 18.33 ^a^

Values with different letters (a, b) in superscript within the same column are significantly different (α < 0.05; Tukey’s test).

Substrate type had a significant effect on the number of primary tunnels after 24 h of release (*F* = 7.75; df = 2, 12; *p* = 0.0069), however, there was no temperature effect (*F* = 1.33; df = 1, 12; *p* = 0.2707) and also no interaction effect (*F* = 0.58; df = 1, 12; *p* = 0.5731). Similarly, substrate type effected the number of branched tunnels (*F* = 10.07; df = 2, 12; *p* = 0.0027) but not the temperature (*F* = 4.17; df = 1, 12; *p* = 0.638) or the interaction of substrate and temperature (*F* = 3.31; df = 2, 12; *p* = 0.0717). The number of branched tunnels was greater in sand or silt loam than in the sandy loam at 22 °C and the number of primary tunnels was greater in sand compared to sandy loam but not the silt loam at 24 h post-release (Table 2).

**Table 2:** Mean number (±SEM) of primary and branched tunnels excavated in 24 h period in three different substrates at two temperature conditions.

Substrate	Mean number of primary tunnels in 24 h		Mean number of branched tunnels in 24 h	
	28 °C	22°C	28 °C	22 °C
sand	2.33 ± 0.33 ^a^	3.33 ± 0.88 ^a^	9.33 ± 3.33 ^a^	18.00 ± 3.60 ^a^
sandy loam	1.00 ± 0.00 ^a^	1.00 ± 0.00 ^b^	4.66 ± 1.20 ^a^	1.66 ± 0.66 ^b^
silt loam	2.00 ± 0.57 ^a^	2.33 ± 0.33 ^ab^	7.33 ± 0.33 ^a^	13.66 ± 2.90 ^a^

Values with different letters (a, b) in superscript within the same column are significantly different (α < 0.05; Tukey’s test).

It is generally agreed that substrate moisture is one of the critical factors that favor foraging activity of subterranean termites. However, there are wide-ranging suggestions when it comes to concluding what is the preferred level of moisture for optimum tunneling activity [2,7,8,10,11]. The rate of tunneling by *C. formosanus* was found to be faster in higher moisture sand than in lower moisture sand only during the middle 3–4 days of the 10 days test period [8]. Similarly, the tunneling rate by *C. frenchi* was reported to be faster in wet sand than in dry sand [7], however, the exact moisture content of the wet sand was not determined. The present data shows that the total tunnel length excavated by *C. formosanus* did not differ between 5% and 15% moisture sand. Our earlier study on sand moisture preference also showed that *C. formosanus* did not preferentially select a particular moisture level among a range of moisture levels from 4 to 24% [11]. This suggests that *C. formosanus* readily gets moisture required for its normal tunneling activity from sand that has as low as 4% moisture. In sandy loam, *C. formosanus *constructed very short tunnels initially but later stopped and very few termites were recovered at the end of the test period. Since both of the substrates had equal amount of moisture by wt., it is conceivable that sandy loam had lower moisture availability compared to sand. However, only moisture availability may not have determined the preference as evident from the fact that 15% moisture sand has more moisture availability than the 5% moisture sand but the termites did not prefer one over the other. Therefore, we suggest that both the texture type and the lower moisture availability could have contributed to make the sandy loam less preferable over sand.

The present results showed that the rate of tunneling and the spatial dispersion of tunnel webs were influenced by the substrate type and ambient temperature. The greater total length of tunnel network in sand than in sandy loam at 12 h or 24 h post-release could primarily be due to the uniformly coarser particle size present in the sand. Termites construct tunnels by picking up soil particles and depositing them elsewhere to clear the way for tunnels [19,20]. Tunnel construction by *C. formosanus* was reported to be faster in uniformly coarse sand (particle size: 0.59–0.84 mm) than in the sand with varied particle size (0.30–2.0 mm) [21]. Although coarse particles would usually be faster to excavate, very coarse particles also could be impenetrable for termites. Studies have shown that sand particle sizes that ranged from 1.2–1.7 mm and 1.7–2.4 mm diameter were impenetrable to *R. hesperus* [22] and *C. formosanus* [23], respectively. As termites pick up individual particles while excavating tunnels, they would have to travel back and forth more often to displace the same volume of substrate with finer particles thus requiring more effort and time to do the same job. Li and Su suggested that the four mouth parts form buccal cavity and load 3–4 sand particles (0.300–0.355 mm) at a time depending upon the size [19]. However, this may not necessarily mean that termites always collect as many fine particles from silt or clay to make the equivalent bulk as a few particles of coarse sand or a single particle of maximum size they can load. In addition, some of the finer particles may drop on the way requiring another trip thus delaying the excavation work. Our observation where longer tunnels were recorded in sand than in the sandy loam or silt loam demonstrates that *C. formosanus* excavates faster in uniform coarse particles than the substrate having finer particle size. 

An efficient search tunnel system would be the one that minimizes the energy expenditure in tunnel excavation and be achieved by minimizing the total length of tunnels from origin to the first food source. Construction of relatively straight and narrow tunnels helps to optimize the time and speed of excavation [24]. The branching frequency and the branch tunnel length play an important role in termite foraging efficiency [25]. Primary tunnels mainly determine how far the foraging area is covered from the origin and the organization of branching system determines how efficient the food search system foraging groups of subterranean termites employ [1]. Construction of a larger number of branched tunnels (secondary, tertiary and quaternary tunnels) helps to exploit the maximum area of the foraging site. In the present experiment, *C. formosanus* built more extensive tunnel webs in sand than in sandy loam as evident from higher number of primary as well as secondary tunnels in both the temperature conditions. We did not determine, however, whether the width and the straightness of the tunnels were some of the possible reasons of greater tunnel length in sand as suggested by Sim and Lee [24]. *C. gestroi* and *H. tenuis* were reported to construct more secondary tunnels at 20 °C and 25 °C than at 15 °C in sand (2). Probably 15 °C is very low temperature for normal activities for the termites. It would be interesting to see how *C. formosanus* tunneling would be impacted at 15 °C. Surprisingly, silt loam, which has a lower proportion of sand than the sandy loam, had greater number of branched tunnels and greater total length of tunnels. We do not think moisture was a limiting factor in sandy loam or silt loam because the moisture content of these substrates was 30% whereas that of the sand was only 15%. We assume that, in addition to particle size and soil moisture, there are some other factors that influence the speed of excavation. Organic matter content was exceptionally high in the sandy loam and pH was slightly higher, however, it is unknown whether these factors have a role to lessen the tunneling activity. An increase in tunneling activity by *C. formosanus* has direct biological relevance as it will increase the likelihood of finding a monitor or bait placed in the nearby areas of infested structures. 

## 3. Experimental Section

### 3.1. Termites

Formosan subterranean termites were collected from a heavily termite-infested area of Brechtel Park in New Orleans, Louisiana in October 2010 using milk crate traps as described in Gautam and Henderson [11]. The termites were maintained in the laboratory (in trash cans containing moist wood) for ~3 months before they were used for the bioassays. 

### 3.2. Preference Tests

**Figure 3:**
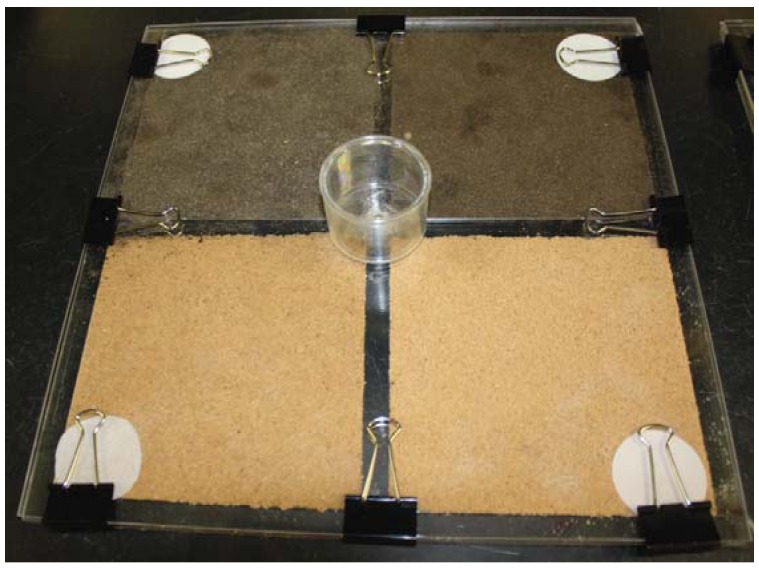
Two dimensional experimental arena for substrate type preference tests.

Bioassay arenas (Figure 3) were prepared according to Hedlund and Henderson [1] with some modification. Each arena consisted of two square Plexiglas^®^ plates (33 by 33 by 0.3 cm) assembled together to make a tunneling chamber 0.3 cm thick. The upper and lower plates were held together with metal binder clips where test substrate was sandwiched in the middle portion separated by 4 narrow edge strips (1.0 by 0.3 by 33 cm or 31 cm). An orifice (0.5 cm diam) was opened with a soldering iron on the central point of the upper plate. The lower plate was divided into 4 equal parts by 4 strips (1.0 by 0.3 by 14.0 cm). These divider strips were glued in a way that an open space was created on the central point along the orifice of the upper plate and four equal gates leading to four parts of the arena were formed. Each part of the arena was filled with one of the 4 treatment substrates: 15% moisture sandy loam, 5% moisture sandy loam, 15% moisture sand and 5% moisture sand, and leveled for uniform compactness. The percent moisture of the test substrates was determined by wt. (the substrate was oven dried until there was no reduction of weight in the subsequent two times and water was added based on wt). Four filter papers (Whatman^®^, 4.2 cm diam) one at each compartment was placed as a food source. A circular acrylic container (called release chamber; size: 5.08 by 3.63 cm, Pioneer Plastics Inc., North Dixon, KY, USA) with the same sized hole on the bottom side was glued to the upper plate so that the two holes (holes of the upper plate and the release chamber) were aligned with each other and the termites from the release chamber could easily get access to the tunneling chamber. We chose sand (fine construction sand, Louisiana Cement Products LLC, Baton Rouge, LA, USA) and sandy loam (collected from backyard of the second author, St. Gabriel, LA, USA) for the substrate preference study as sand represented the low organic matter substrate with uniformly coarser particles and sandy loam represented the high organic matter substrate with finer particle size (Table 3). Two moisture levels were chosen based on our experience on conducting laboratory experiments. Five percent moisture represented the lower range of sand moisture for most laboratory experiment and 15% represented the upper range. Soil compaction was recorded by measuring the wet bulk density of test substrates in the foraging arena, which were as followed: Sand 2.33 g/cm^3^, sandy loam 1.22 g/cm^3 ^and silt loam 1.32 g/cm^3^.

One hundred termites (88 workers, 10 soldiers and 2 nymphs; workers were externally undifferentiated individuals developed to at least third instar) were released in the releasing chamber which contained 10 g of mixture of both the substrates (sandy loam and sand) moistened to 15% by wt. Nymphs were included in the tests since the field collected groups were composed of workers, soldiers and nymphs. The test was repeated 5 times.

Number of termites present in each substrate was recorded after 24 h. To record the number of termites present in each compartment, the arena was placed on a scanner and the individuals were counted from the top while the bottom was scanned at the same time so that duplication in counting was avoided. The scanned image transferred on computer was used to count the termites seen from the bottom part of the arena. Since the thickness of the substrate was only 0.3 cm, all termites in the tunnels were visible either from the top or from the bottom of the foraging chamber. Termites not seen on the foraging chamber from either side were presumed to be present in the releasing chamber and were not included in the analysis. Length of the tunnels was measured with the help of a string overlaid on the tunnels. Only one measurement was taken of the tunnels that were visible from both the sides of the foraging chamber, otherwise separate measurements were taken. Total length of the tunnels in each substrate was then calculated by adding the lengths of primary and branched tunnels (branched tunnels included both secondary and the tertiary tunnels). 

### 3.3. Tunneling Tests

The bioassay arena consisted of a similar structure as described in the preference tests except that the release chamber was fitted to one edge of the square tunneling chamber, a filter paper placed on the opposite edge of the chamber and the foraging arena was filled with only one type of substrate, so, there were no dividers in the arena. One more substrate type, silt loam (collected from Ben–Hur Research Station, Baton Rouge, LA, USA) was added for tunneling tests, which had higher organic matter than the sand but lower than the sandy loam and had the finest particle of all the three substrates (Table 1). Sand was moistened to 15% by wt. and sandy loam and silt loam to 30% by wt. These moisture levels were chosen to approximately equate the proportion of their saturation level based on their moisture holding capacity, as sand gets saturated at ~28% moisture whereas the other two substrates get saturated at ~55%. Two temperature conditions, 22 °C and 28 °C were chosen because the former temperature represents the most common laboratory room temperature and the latter represents the temperature usually maintained in the incubator for most termite bioassays. The release chamber contained 10 g of the same substrate as in the foraging chamber.

One hundred termites (88 workers, 10 soldiers and 2 nymphs; workers were externally undifferentiated individuals developed to at least the third instar) were introduced in the release chamber and the arena was placed undisturbed either in the incubator set to 28 °C or in the laboratory room having a temperature at 22 °C. There were 3 replications for each temperature and substrate type. Tunneling was recorded after 14 h and 24 h of release. Different colored permanent marker pens were used to trace the tunnels made at each time period. The number of primary and branched tunnels was recorded and compared among the tested substrates. The total length of the tunnels in the arena was calculated as described in the preference tests. 

**Table 3:** Characteristics of substrates used in experiments.

Sample ID	Organic matter (%)	Particle size distribution (%)			Textural class
		Sand	Silt	Clay	
Ben Hur Soil	6.56	20.6	53.1	26.3	Silt Loam
St. Gabriel Soil	15.09	68.4	22.8	8.8	Sandy Loam
Sand	0.09	99.1	0.6	0.3	Sand

### 3.4. Statistical Analysis

Data analysis was done with SAS 9.1 software [26]. Tunnel volumes and number of primary and branched tunnels were subjected to ANOVA using a generalized linear model. Location count data were square root transformed to improve normality and analyzed using ANOVA. Untransformed data are reported in the text. Multiple comparisons were done using Tukey’s honestly significance difference at α = 0.05.

## 4. Conclusions

Understanding the role of substrate types, moisture levels and temperatures on the search tunnel formation by subterranean termites is important to optimizing the control strategies. We showed that *C. formosanus* preferentially selected a substrate with uniform coarser particles (sand) for tunnel excavation and aggregation over the substrate with heterogeneous and finer particles (sandy loam). The rate of tunneling and the spatial dispersion of tunnel webs were also greater in sand than either sandy loam or silt loam. However, termite aggregation and tunneling were not different based on whether the sand had 5% or 15% moisture level. In addition to particle size and soil moisture, some other factors such as organic matter and pH may have influenced the speed of excavation which needs further investigations. Preference for tunneling activity by subterranean termites has direct implications in termite management, particularly the termite baiting system. A successful termite baiting relies on how quickly the monitors are hit by the subterranean termites. The present results suggest that the likelihood of finding a bait station placed in the nearby areas of infested structures increases when the substrate in the surroundings is primarily sandy compared to sandy loam or silt. 
